# Beneficial Effects of THSG on Acetic Acid-Induced Experimental Colitis: Involvement of Upregulation of PPAR-γ and Inhibition of the Nf-Κb Inflammatory Pathway

**DOI:** 10.3390/molecules16108552

**Published:** 2011-10-12

**Authors:** Cheng Zeng, Jun-Hua Xiao, Mu-Jun Chang, Jia-Ling Wang

**Affiliations:** Department of Pharmacology, Tongji Medical College, Huazhong University of Science and Technology, Wuhan 430030, China; Email: zengcheng1110@yahoo.com.cn (C.Z.); 84149528@qq.com (J.-H.X.); 450755782@qq.com (M.-J.C.)

**Keywords:** 2,3,5,4’-tetrahydroxystilbene-2-*O*-beta-D-glucoside, colitis, PPAR-γ, NF-κB

## Abstract

The polyphenolic compound 2,3,5,4′-tetrahydroxystilbene-2-*O*-beta-D-glucoside (THSG) has been shown to possess anti-inflammatory effects. Here, we examined the effects of THSG on experimental mice with colitis induced by acetic acid and whether the underlying mechanisms were associated with the PPAR-γ and NF-κB pathways. Mice were randomized into six equal groups: normal, colitis model, THSG (10, 30, 60 mg·kg^−1^) and mesalazine. The mice were administered 10, 30, 60 mg·kg^−1^ THSG or 100 mg·kg^−1^ mesalazine or saline once daily by intragastric administration for 7 days after induction of colitis by acetic acid irrigation. THSG dramatically attenuated acetic acid-induced colon lesions, including reversing the body weight loss and improving histopathological changes. THSG apparently decreased the increase of malondialdehyde (MDA) which is a marker of lipid peroxidation. THSG appears to exert its beneficial effects on acetic acid-induced experimental colitis through upregulation of PPAR-γ mRNA and protein levels and inhibition of the NF-κB pathway, which in turn decreases the protein overexpression of the downstream inflammatory mediators TNF-α, IL-6 and COX-2. The effect of THSG 60 mg·kg^−1^ on PPAR-γ mRNA expression was higher than that of mesalazine. THSG may thus be a promising new candidate or lead compound for the treatment of IBD.

## 1. Introduction

Inflammatory bowel diseases (IBD), including Crohn’s disease and ulcerative colitis, have a high incidence in industrialized nations and severely affect the life quality of patients. They are chronic and relapsing inflammatory disorders of the gastrointestinal tract, defined by clinical characteristics such as diarrhea, abdominal pains, weight loss and nausea and by pathological features such as a loss of mucosal integrity and inflammatory cell infiltration [1]. The conventional therapeutic strategy is to use anti-inflammatory agents such as mesalazine, corticosteroids and immunosuppressive agents which non-specifically reduce the immune response and inflammation. In the last two decades, and thanks to a better understanding of the key role of the components involved in the inflammatory pathway, the inflammatory mediators TNF-α, IL-6, COX-2 and their upstream signal regulator NF-κB have become new promising anti-inflammatory therapy targets for the treatment of IBD [2]. Among the steps of the complex inflammatory response in the IBD process, activation of NF-κB leads to NF-κB dimers (p65 and p50) that translocate to the nucleus to promote transcription of the pro-inflammatory mediators TNF-α, IL-6 and COX-2, which then result in a series of inflammatory cascade responses [3,4]. PPAR-γ, a member of the nuclear hormone receptor superfamily, could inhibit activation of NF-κB through several mechanisms and then repress NF-κB-mediated transcription of pro-inflammatory cytokines [5]. Activation of PPAR-γ could potentially reduce the severity of IBD by inhibiting excessive immunoinflammatory responses [6,7,8,9]. PPAR-γ was shown to be involved in the activity of mesalazine, the standard first-line treatment for IBD patients with mild to moderately active ulcerative colitis [10], thus suggesting that colonic PPAR-γ could be a better therapeutic target in humans suffering from IBD [6,11,12,13]. 

**Figure 1:**
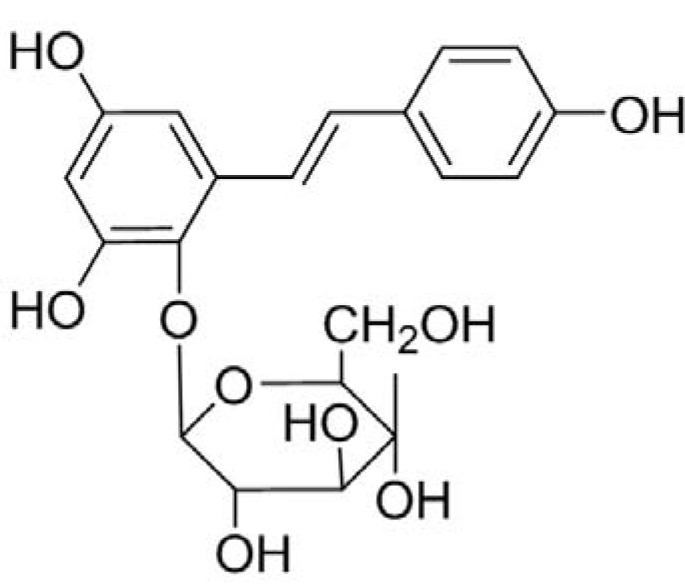
Chemical structure of THSG.

*Polygonum multiflorum Thunb* (PM) is a traditional Chinese medicinal herb that has been used for thousands of years as a tonic and anti-aging agent and also used effectively to prevent graying, to treat skin depigmentation diseases and lubricate the intestine. 2,3,5,4′-Tetrahydroxystilbene-2-*O*-beta-D-glucoside (THSG, Figure 1), extracted from the rhizome of PM, has been demonstrated to possess beneficial effects on atherosclerosis [14,15], Alzheimers’ disease [16] and inflammation [15,17]. THSG has a polyphenolic structure, similar to that of resveratrol, and may act on experimental colitis by inhibiting production of inflammatory mediators and attenuating the inflammatory response [18,19,20]. Our previous data suggested that THSG could exert protective effects on experimental colitis through alleviating oxygen and nitrogen free radicals and down-regulating iNOS expression [21].

In this paper we focused on the effects of THSG on PPAR-γ and the NF-κB-induced inflammatory mediators TNF-α, IL-6, COX-2 in an mouse experimental model of colitis induced by acetic acid, with the aim of revealing the underlying mechanisms of the beneficial effects of THSG on colitis and provide evidence that THSG may be a promising new candidate or lead compound for the treatment of IBD.

## 2. Results and Discussion

### 2.1. Results

#### 2.1.1. Beneficial Effects of THSG on Acetic Acid-Induced Colitis in Mice

**Figure 2:**
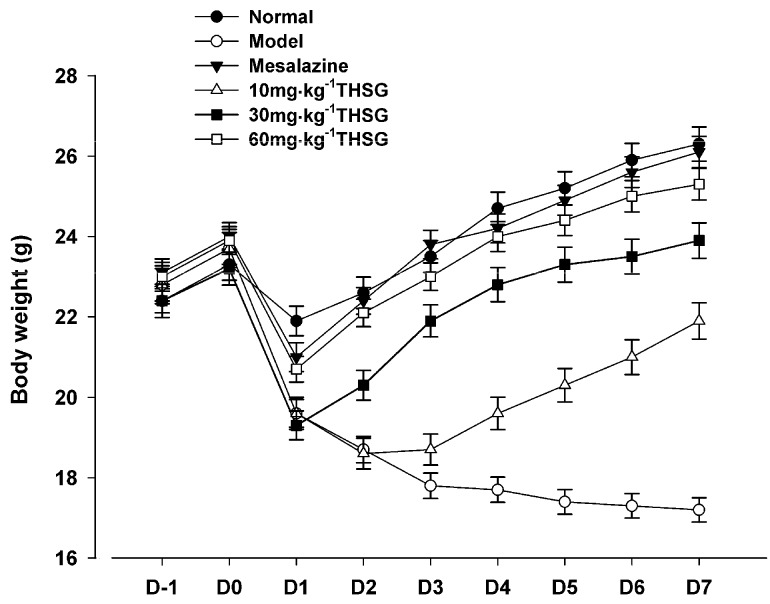
Effect of THSG on mice body weight loss caused by experimental colitis induced by acetic acid. Values are means ± SEM (n = 12 for each group).

To assess the beneficial effects of THSG on acetic acid-induced colitis in mice and confirm whether the colitis model had been successfully established, we monitored the changes in body weight and colon histological appearance of the experimental animals. As shown in Figure 2, seven days after establishing the model, the body weight of the treated mice deceased gradually, compared with the normal (untreated) mice. Mesalazine, a positive drug control, significantly reversed the loss of the body weight, while 10, 30, 60 mg·kg^−1^ THSG could also amelliorate the loss of body weight in a dose-dependent manner. THSG 60 mg·kg^−1^ enhanced the body weight to levels similar to those of the mice in the normal and mesalazine groups.

**Figure 3:**
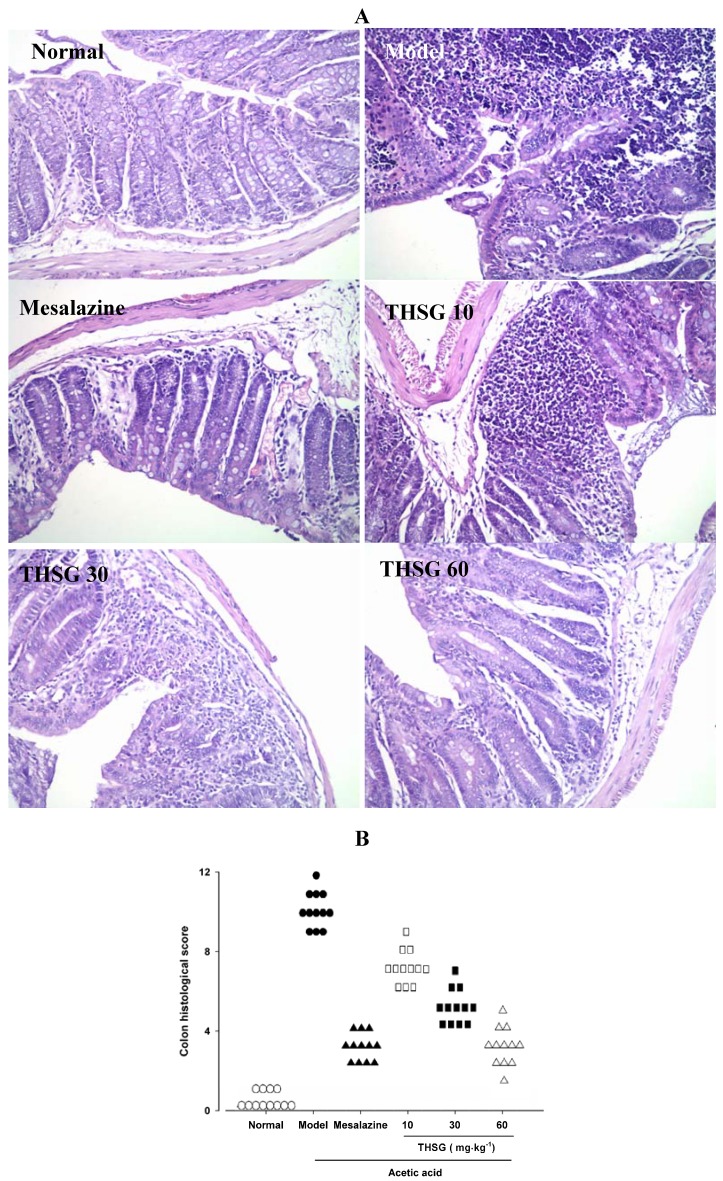
Beneficial effects of THSG on experimental colitis induced by acetic acid in mice (**A**) Representative slides of haematoxylin and eosin (H & E) stain (magnification 200 ×). (**B**) The histological scores derived from histopathologic evaluation of colon in mice. (n = 12 for each group).

The colon morphological studies showed that acetic acid induced serious damage to the mucous glands and inflammatory cell infiltration. Mesalazine could significantly improve the inflammatory response induced by acetic acid. THSG 10, 30, 60 mg·kg^−1^ could also dose-dependently reduce colitis damage induced by acetic acid. The histological score in the THSG 60 mg·kg^−1^ group was as low as that seen in the mesalazine group (Figure 3).

#### 2.1.2. Effect of THSG on MDA Levels

**Figure 4:**
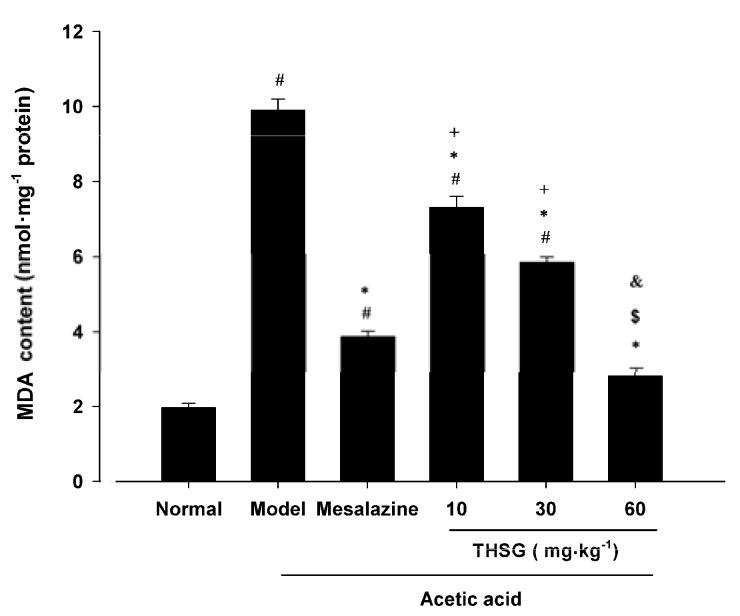
Effect of THSG on MDA content in colonic tissues of mice treated with acetic acid. Values are means ± SEM (n = 12 for each group). # *p <* 0.05 *versus* Normal group; * *p* < 0.05 *versus* Model group; + *p* < 0.05 *versus* Mesalazine group; $ *p* < 0.05 *versus* THSG 10 group; & *p* < 0.05 *versus* THSG 30 group.

MDA is a lipid peroxidation marker that plays a key role in tissue damage. A previous study had shown that a significant increase of MDA ocurred in the inflamed colonic tissues of mice challenged with acetic acid [21]. MDA levels increased significantly from 1.97 ± 0.12 nmol·mg^−1^ to 9.90 ± 0.3 nmol·mg^−1^ in the model group. THSG 10, 30, 60 mg·kg^−1^ attenuated the MDA levels in a dose–dependent manner and the corresponding MDA levels were decreased to 7.3 ± 0.3, 5.85 ± 0.14, 2.81 ± 0.21 nmol·mg^−1^, respectively. There was no difference between the 60 mg·kg^−1^ THSG and the mesalazine group (Figure 4).

#### 2.1.3. Effects of THSG on Inflammatory Mediators TNF-α, IL-6 and COX-2

**Figure 5:**
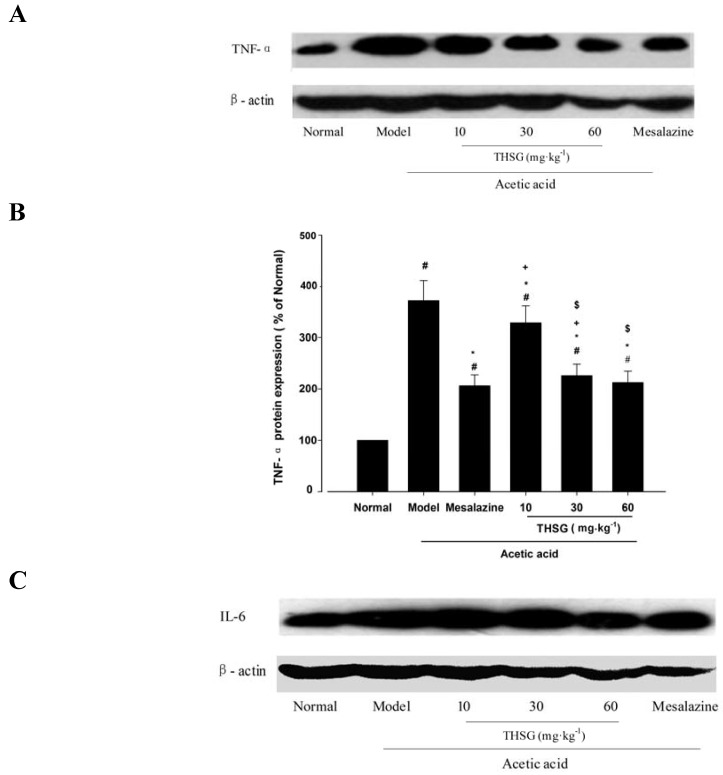

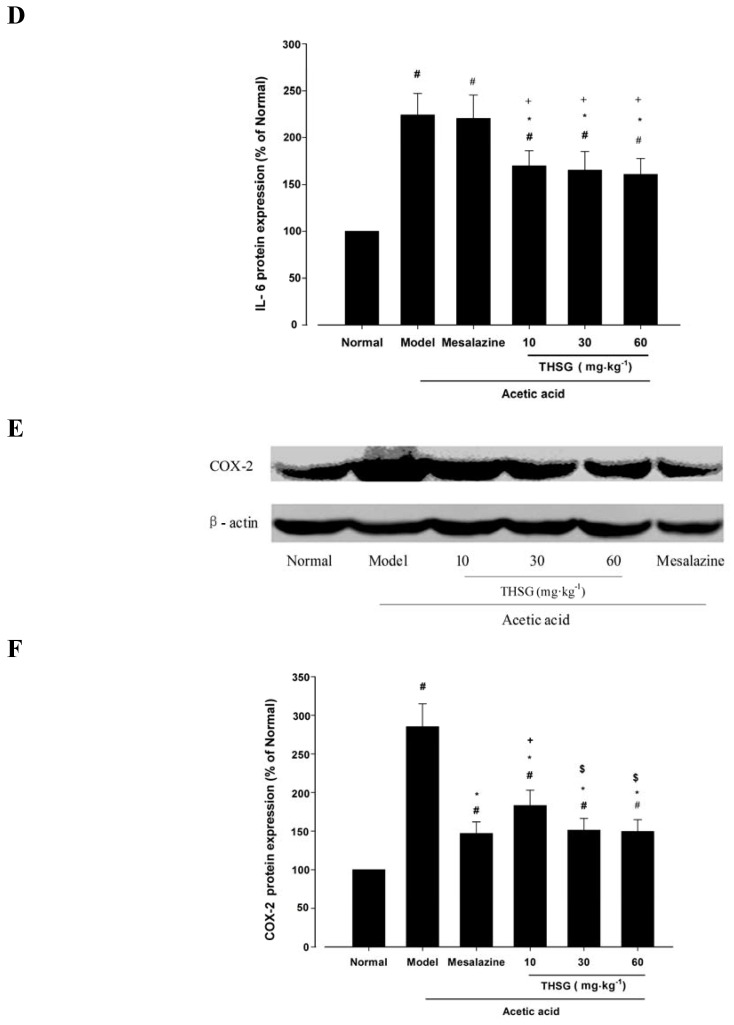
Effects of THSG on expression of the inflammatory mediators TNF-α, IL-6 and COX-2 in colonic tissues of mice challenged by acetic acid assessed by Western blot analysis (**A**) Immunoreactive bands of TNF-α using specific antibody. Actin was used as an internal control. (**B**) Histogram representing the quantitative analysis of TNF-α level normalized to actin protein. (**C**) Immunoreactive bands of IL-6 using specific antibody. (**D**) Histogram representing the quantitative analysis of IL-6 level normalized to actin protein. (**E**) Immunoreactive bands of COX-2 using specific antibody. (**F**) Histogram representing the quantitative analysis of COX-2 level normalized to actin protein. Date in B, D and F are expressed as means ± SEM (n=6 for each group). # *p <* 0.05 *versus* Normal group; * *p* < 0.05 *versus* Model group; + *p* < 0.05 *versus* the Mesalazine group; $ *p* < 0.05 *versus* THSG 10 group; & *p* < 0.05 *versus* THSG 30 group.

To evaluate the anti-inflammatory role of THSG in the colon, the levels of colonic molecular markers of inflammation such as TNF-α, IL-6 and COX-2 were measured by Western blot analysis using specific antibodies against these markers. As shown in Figure 5A and B, TNF-α expression was markedly elevated (371.9 ± 39.2% of normal) in the model group, while mesalazine decreased the TNF-α overexpression. THSG 10, 30, 60 mg·kg^−1^ could also dose-dependently decrease the acetic acid-induced TNF-α overexpression in colonic tissues, and 60 mg·kg^−1^ THSG reversed the overexpression of TNF-α to 212.6 ± 22.1% (*p* > 0.05 *versus* normal group). 

IL-6 was over-expressed in the colon after the acetic acid treatment. THSG 10, 30, 60 mg·kg^−1^ inhibited the overexpression of IL-6 to 170.0 ± 16.6%, 165.2 ± 20.1% and 160.6 ± 17.3%, which are lower values than those achieved with mesalazine treatment. The data are shown in Figure 5C and D.

COX-2 expression was significantly increased to 285.1 ± 30.5% of normal in the model group (*p* < 0.05 *versus* normal group). Mesalazine could inhibit the overexpression of COX-2 to 146.9 ± 15.6%. COX-2 protein levels were shown to 183.1 ± 20.4%, 151.2 ± 15.7% and 149.5 ± 15.2% in colonic tissues treated with THSG 10, 30, 60 mg·kg^−1^ for seven days (Figure 5E and F).

#### 2.1.4. Effect of THSG on NF-κB Expression

**Figure 6:**
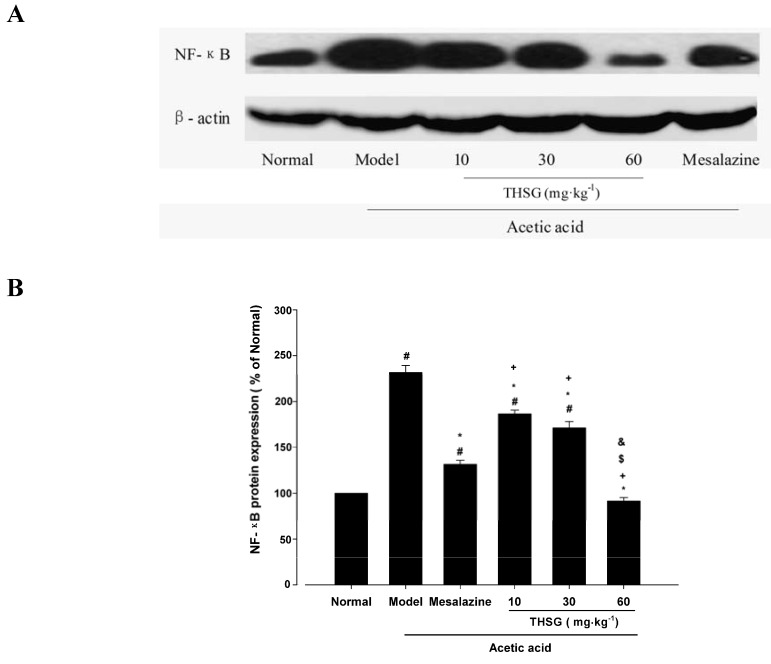
Effect of THSG on the expression of NF-κB in colonic tissues of mice treated with acetic acid assessed by Western blot analysis. (**A**) Immunoreactive bands of NF-κB and actin using specific antibody. Actin was used as an internal control. (**B**) Histogram representing the quantitative analysis of NF-κB level normalized to actin protein. Data are expressed as means ± SEM (n=6 for each group). # *p <* 0.05 *versus* Normal group; * *p* < 0.05 *versus* Model group; + *p* < 0.05 *versus* Mesalazine group; $ *p* < 0.05 *versus* THSG 10 group; & *p* < 0.05 *versus* THSG 30 group.

NF-κB is an important transcriptional regulator of inflammatory mediators at the gene level and also a convergence point in the inflammatory signal network, so we examined the effect of THSG on NF-κB p65 expression in the acetic acid-induced experimental colitis model. As shown in Figure 6, NF-κB p65 expression was dramatically increased in the model group, compared with the normal group (240.9 ± 25.7% of normal, *p* < 0.05 *versus* normal group). Mesalazine could decrease the elevated NF-κB p65 expression to 75.4 ± 9.9%, which is higher than the normal group. THSG 10, 30, 60 mg·kg^−1^ could inhibit the increase of NF-κB p65 expression in mice colonic tissues induced by acetic acid to 148.5 ± 16.1%, 104.9 ± 11.2% and 30.1 ± 11.4%, respectively. The inhibitory effect of THSG 60 mg·kg^−1^ on NF-κB p65 overexpression seemed better than the effect of mesalazine (*p* < 0.05 *versus* mesalazine group).

#### 2.1.5. Effect of THSG on PPAR-γ Expression

**Figure 7:**
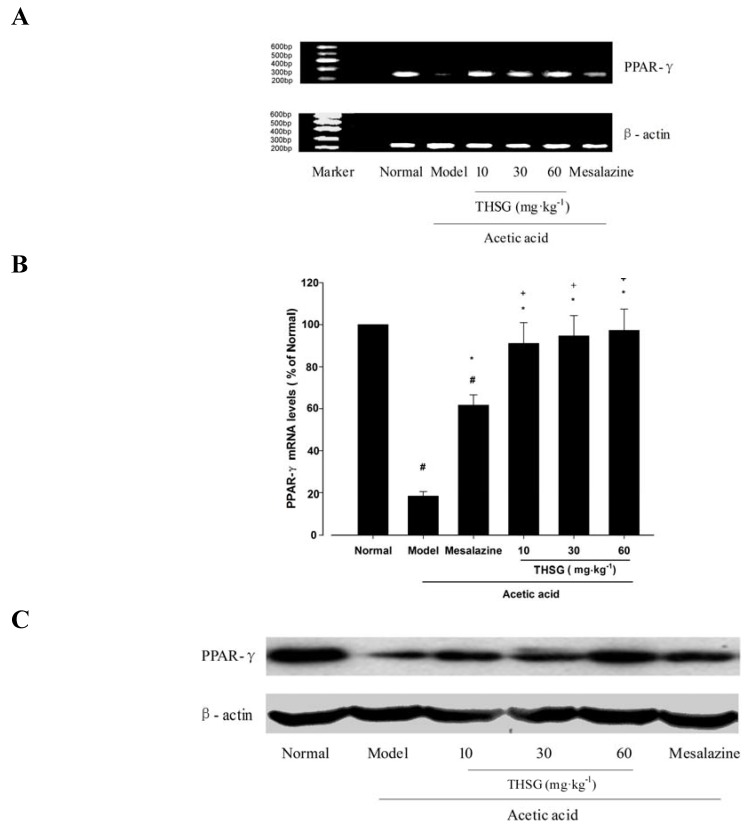

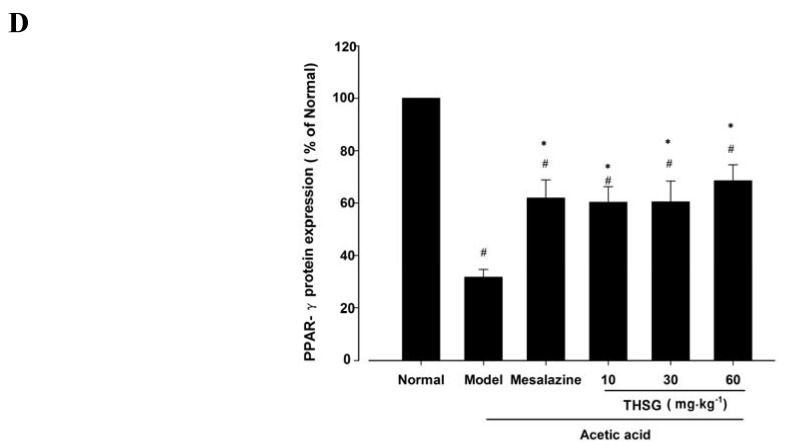
Effects of THSG on PPAR-γ mRNA and protein expression levels in colonic tissues treated with acetic acid. (**A**) RT-PCR analysis of products PPAR-γ and β-actin. (**B**) Histogram representing the quantitative analysis of PPAR-γ mRNA levels compared to the control after normalization to β-actin. (**C**) Immunoreactive bands of PPAR-γ and actin using specific antibody. PPAR-γ protein expression was assessed by Western blot analysis. Actin was used as an internal control. (**D**) Histogram representing the quantitative analysis of PPAR-γ level normalized to actin protein. Data in B and D are expressed as means ± SEM (n = 6 for each group). # *p <* 0.05 *versus* Normal group; * *p* < 0.05 *versus* Model group; + *p* < 0.05 *versus* Mesalazine group; $ *p* < 0.05 *versus* THSG 10 group.

PPAR-γ, expressed at high levels in the colonic epithelium, plays an important role in the regulation of colon inflammation [6]. To explore whether PPAR-γ was involved in the anti-inflammatory effect of THSG on mice with experimental colitis, we studied the PPAR-γ expression in colonic tissues at the mRNA and protein levels. As shown in Figure 7, PPAR-γ mRNA and protein were highly expressed in normal mice colonic tissues and was robustly reduced to 18.5 ± 2.6%, 31.7 ± 3.6% of normal in the model group (*p* < 0.05 *versus* normal group). Mesalazine could reverse the decreased expression of PPAR-γ mRNA and protein levels induced by acetic acid and THSG 10, 30, 60 mg·kg^−1^ could enhance PPAR-γ mRNA and protein expression. The effect of THSG 60 mg·kg^−1^ on PPAR-γ expression was higher than that of mesalazine at the mRNA level (*p* < 0.05 *versus* mesalazine group).

### 2.2. Discussion

Exciting results were found in this study. THSG, a polyphenolic compound from PM, had a pretty good effect on experimental colitis induced by acetic acid as judged through the significant reversal of body weight loss and attenuation of histological lesions observed in experimental animals. THSG countered the increased content of MDA and lowered the protein overexpression of the inflammatory mediators TNF-α, IL-6 and COX-2, and the nuclear transcript regulator NF-κB. THSG significantly enhanced the PPAR-γ mRNA and protein levels. The effect of THSG 60 mg·kg^−1^ on PPAR-γ mRNA expression was higher than that of mesalazine.

Cumulative evidences have shown that overproduction of inflammatory mediators released by effector leukocytes determines the elicitation and maintenance of the disease [22]. In the coordinated network of the inflammatory response, TNF-α plays an important signaling role in the subsequent inflammation cascade — after secretion by activating monocytes and macrophages, TNF-α induces intestinal immune cells to elaborate prostaglandins and proteases, as well as other inflammatory and chemotactic cytokines. An obviously enhanced expression of TNF-α was found in IBD, and the important role of TNF-α in the genesis of these diseases was also confirmed [2]. Infliximab, a chimeric anti-TNF-α monoclonal antibody, has become a standard therapy for the treatment of inflammatory Crohn’s disease in Europe. In present study, TNF-α was downregulated at the protein level in colonic tissues treated with THSG.

IL-6 is a pleiotropic cytokine with a central role in immune regulation and inflammation. Overproduction of IL-6 has been found in many types of colitis, in which it exerts its proinflammatory effects largely through activation of signal transducers and as an activator of transcription-3 [23]. IL-6–activated macrophages and colon epithelial cells secrete inflammatory cytokines, including TNF-α and IL-1β, which contribute to the development of colitis [24]. Our results also showed that THSG could repress the expression of IL-6 in colonic tissues challenged by acetic acid.

COX-2, the inducible form of the enzyme, is markedly increased in inflamed tissue in IBD patients. Upon the recognition of bacterial products in the intestinal lumen by the Toll-like receptors (TLRs), in particular TLR4, COX-2 expression is induced by transcription factors such as NF-κB. Then the induced COX-2 regulates the production of prostaglandins, the central mediators of inflammation [25], so COX-2 plays a key role in the regulation of the inflammatory response. Our data showed that THSG could obviously inhibit the expression of COX-2 in colonic tissues of mice with experimental colitis.

Overproduction of inflammatory mediators (TNF-α, IL-6, COX-2) in IBD can lead to a positive inflammatory feedback loop which can produce toxic peroxide anions, proteases and oxygen/nitrogen radicals and cause indiscriminate tissue damage [2]. Reactive oxygen species (ROS) also play a pathogenic role in IBD [26,27]. Moreover, there is a crosstalk between inflammation and oxidative stress [28,29]. In pathological contexts, they can provide positive feed-back leading to an uncontrolled pathological process. MDA, as a marker of lipid peroxidation, can interact with DNA and proteins and then lead to pathological changes [30]. Our results also showed that THSG could significantly reduce the MDA in colonic tissues of mice treated by acetic acid, accordingly break the feedback loop and attenuate the lesion of colonic tissues.

In view of the fact that THSG could obviously and attenuate the MDA levels and the expression of the inflammatory mediators TNF-α, IL-6 and COX-2, we then measured their upstream regulatory factor NF-κB. 

NF-κB is a pleiotropic transcription factor that regulates inducible gene expression under both physiological and pathological conditions. The role of NF-κB in mediating intestinal inflammation has been established in human disease and animal inflammatory models. NF-κB, the inflammatory mediator nuclear transcription factor, can be activated by pro-inflammatory cytokines and endotoxins and then translocate to the nucleus to promote transcription of inflammatory mediators, such as TNF-α, IL-6 and COX-2 [31], so NF-κB is a key component controlling the intensity, duration and consequences of IBD. Our results demonstrated that THSG could markedly reduce the expression of NF-κB in colon tissues of mice with model colitis.

Owning to the fact that the expression and activation of NF-κB could be modulated by PPAR-γ through several mechanisms, including direct interactions with NF-κB, nucleo-cytoplasmic redistribution of the p65 subunit of NF-κB and interactions with transcriptional co-repressors of NF-κB [32], we measured the expression of PPAR-γ at mRNA and protein levels in colonic tissues of mice with colitis induced by acetic acid.

PPAR-γ, a member of the nuclear hormone receptor superfamily expressed on T-cells, macrophages, dendritic cells and epithelial cells, is an important regulator maintaining the balance of mucosa immunity. Accumulative evidence has shown a significant association between deficiency of PPAR-γ and IBD [33,34]. In addition, activation of PPAR-γ could attenuate the inflammation in the gut [35]. These results suggested that colonic PPAR-γ may be a promising therapeutic target in humans suffering from IBD. Our data indicated that THSG could significantly increase PPAR-γ expression at both the mRNA and protein levels in colonic tissues of mice with experimental colitis.

It is worth mentioning that Avandia, an agonist of PPAR-γ, has been associated with an increase in the risk of myocardial infarction and death from cardiovascular causes [36]. THSG has shown beneficial effects on cardiovascular diseases [14,15,37]. PM has been used for thousands of years as Chinese traditional medicine and THSG possesses a good safety profile demosntrated through acute and chronic toxicity testing [38].

## 3. Experimental

### 3.1. Materials

THSG (purity >98% by HPLC) was provided by the Phytochemistry Laboratory, Department of Pharmacology, Tongji Medical College, Huazhong University of Science & Technology (China). Mesalazine was donated by Yuan Cheng Co Ltd (Wuhan, China). Acetic acid was purchased from the China National Pharmaceutical Group Corporation Shanghai Chemical Reagent Company (Shanghai, China).

### 3.2. Experimental Animals

Male Kunming mice weighing 20–25 g were obtained from the Experimental Animal Center of Tongji Medical College, Huazhong University of Science and Technology (permit numbers: SCXK (Hubei) 2004-0007). All mice were treated in accordance with the *Guide for the Care and Use of Laboratory Animals* published by the U.S. National Institutes of Health.

### 3.3. Experimental Protocol

Seventy-two mice were equally randomized into six groups: normal, colitis model, THSG (10, 30, 60) and mesalazine. In THSG and mesalazine groups, the mice were treated with 10, 30, 60 mg·kg^−1^ THSG or 100 mg·kg^−1^ mesalazine once daily by intragastric administration (ig) for 7 days, after induction of colitis by acetic acid. In the normal and model groups, the mice were given an equal volume of saline.

### 3.4. Preparation of Colitis Mice Model Induced by Acetic Acid

The preparation of the colitis mice model was described in our previous report [21]. In brief, mice were kept in a room under the controlled temperature (25 °C) with a light–dark cycle of 12 h each day. They were deprived of food for 12 h and allowed free access to water before preparation. Mice were anaesthetized by an intraperitoneal injection of 3.5 mL·kg^−1^ 10% chloral hydrate. Then a medical polyurethane cannula (external diameter 2 mm) was inserted into 3 cm proximal to the anus. After lavage with 5% soapy water (0.2 mL) and saline (0.2 mL), the rectum was irrigated with 5% acetic acid (0.1 mL) through the cannula. The mice were kept in a head-down position for approximately 20 sec to prevent leakage of the intracolonic acetic acid. Finally, the rectum was washed with saline (0.2 mL). In the normal group, animals were treated with saline (0.1 mL) instead of acetic acid.

### 3.5. Evaluation of Colitis Damage

After being treated for 7 days, the mice were euthanized by an intraperitoneal injection of sodium pentobarbital. The colon tissues near 1 cm proximal from anus were immediately fixed in 10% formalin solution and embedded in paraffin. The paraffin-embedded colon tissues were sectioned and stained with hematoxylin and eosin (HE). Then, the stained tissues were observed with a light microscope (Olympus BX40, Tokyo, Japan) at 200 × magnification. The severity of colitis was evaluated in a blinded way by a previously reported histological scoring system [21,39], whereby inflammatory response was graded according to the extent of destruction of normal mucosal architecture (0 = normal; 1 = mild; 2 = moderate; 3 = extensive damage), presence and degree of cellular infiltration (0 = normal; 1 = mild; 2 = moderate; 3 = transmural infiltration), extent of muscle thickening (0 = normal;1 = mild; 2 = moderate; 3 = extensive thickening), presence or absence of crypt abscesses (0 = absent; 1 = present), and the presence or absence of goblet cell depletion (0 = absent; 1 = present).

### 3.6. Measurement of MDA Level

The frozen colon tissues were weighed and homogenized in 50 mmol·L^−1^ phosphate buffer (pH 7.4) in an ice bath. Then malondialdehyde (MDA) level was measured by colourimetric analysis using 722 spectrophotometer with the appropriate detection kit (Jiancheng, Nanjing, China), and expressed as nmol·mg^−1^ protein.

### 3.7. Western Blot Analysis

Isolated frozen colon tissues were homogenized in the cell lysis buffer (50 mM Tris–HCl, pH 7.4, 100 mM NaCl, 1% NP-40, 5 mM EDTA, 20 mM NaF, 1 mM PMSF, 3 mM Na_3_VO_4_ and protease inhibitors). Protein concentration was determined by the BCA protein assay kit (Pierce Biotechnology, Rockford, IL, USA). Protein samples (40 μg) were separated by 10% SDS–PAGE, and then transferred to PVDF membranes. After being blocked with 5% nonfat milk in TBS-T for 1 h, the membranes were incubated overnight at 4 °C with different primary antibodies specific for TNF-α, IL-6, COX-2 and NF-κB p65 (Santa Cruz Biotechnology), PPAR-γ (Cell Signaling Biotechnology, Boston, MA, USA) and β-actin (Upstate Biotechnology, New York, NY, USA). Bound antibody was detected by HRP conjugated anti-mouse IgG or anti-rabbit IgG. Finally, immunoreactive bands were visualized with ECL reagent. The intensity of bands was quantified by densitometry. The expression levels of TNF-α, IL-6, COX-2, NF-κB p65 and PPAR-γ were normalized to β-actin expression level.

### 3.8. Reverse Transcription Polymerase Chain Reaction (RT-PCR)

Total RNA was extracted from colon using Trizol reagent (Gibco BRL, Gaithersburg, MD, USA) according to the manufacturer's instructions. Complementary DNA was prepared by SuperScript First-Strand Synthesis system for RT-PCR using oligo(dT) as primer. The sense and anti-sense primer sequences used were: PPAR-γ: 5’-GACCACTCGCATTCCTTT-3’; 5’-CCACAGACTCGGCACTCA-3’; β-actin: 5’-CTGT CCCTGTATGCCTCTG-3’; 5’-ATGTCACGCACGATTTCC-3’. The primers set yielded PCR products of 266 bp and 218 bp for PPAR-γ and β-actin, respectively. Thirty-five cycles of amplification were performed at 95 °C for 2 min, then at 94 °C for 30 sec, at 56 °C for 30 sec, and at 72 °C for 40 sec, with a final extension at 72 °C for 5 min using the GeneAmp PCR System 2400 (PA). The PCR products were electrophoresed in 2% agarose gel, and observed by SYBR Green I staining and UV irradiation. The gel photographs were scanned with a computerized densitometer (SYNGENE, London, UK). The ratio of PPAR-γ to β-actin was calculated.

### 3.9. Statistical Analysis

Results are expressed as means ± SEM. Statistical comparisons among groups were analyzed using one-way ANOVA followed by Turkey-Kramer multiple comparison test with a statistical significance of *p* < 0.05.

## 4. Conclusions

In conclusion, the findings provided support for the fact that THSG exerts beneficial effects on acetic acid-induced experimental colitis through upregulation of PPAR-γ and inhibition of the NF-κB pathway, which decreases the expressions of the downstream inflammatory mediators TNF-α, IL-6 and COX-2 and MDA content. It is also suggested that THSG may be a promising new candidate or lead compound for the treatment of IBD.
